# Effect of freshwater on plant species diversity and interspecific associations in coastal wetlands invaded by *Spartina alterniflora*

**DOI:** 10.3389/fpls.2022.965426

**Published:** 2022-09-21

**Authors:** Zhiguo Dou, Lijuan Cui, Wei Li, Yinru Lei, Xueyan Zuo, Yang Cai, Rui Yan

**Affiliations:** ^1^Institute of Wetland Research, Chinese Academy of Forestry, Beijing, China; ^2^Beijing Key Laboratory of Wetland Services and Restoration, Beijing, China; ^3^Institute of Ecological Conservation and Restoration, Chinese Academy of Forestry, Beijing, China; ^4^Yancheng Milu Institute, Jiangsu Dafeng Père David's Deer National Nature Reserve, Yancheng, China

**Keywords:** coastal wetland, invasive plants, biodiversity, freshwater, restoration

## Abstract

Plant invasions in coastal wetlands lead to the degradation of native vegetation; the introduction of freshwater in coastal wetlands would prevent the spread of invasive plants and facilitate the restoration of native vegetation. In this study, we evaluated the effects of freshwater on plant communities in the coastal wetlands of Yancheng, China, invaded by *Spartina alterniflora* Loisel. Two field investigations were conducted in 2008 and 2018 before and after the introduction of freshwater (started in 2011). The characteristics of plant communities were subjected to hierarchical cluster analysis and compared using several diversity indices. In addition, differences in habitat community composition and interspecific relationships of dominant species were analyzed. The results showed that *S. alterniflora* reduced the overall species diversity in the region. Plant species diversity increased after freshwater was introduced into the study site when compared to the areas without freshwater introduction. The introduction of freshwater caused a shift often changes in the interspecific relationships between *Suaeda salsa* (L.) Pall. and other species. The intensified invasion of *S. alterniflora* changed the interspecific relationship of native halophytes from negative to positive. Although freshwater effectively inhibited further invasion of *S. alterniflora*, it also increased the risk of expansion of the glycophytes in the community. The results of this study highlight the need for early intervention for restoration of coastal wetlands, preservation of biodiversity, and management of plant resources.

## Introduction

Biological invasions drive global changes in ecosystems (Vitousek, 1997; Mooney and Hobbs, 2000), which cause huge economic losses (Pimentel et al., 2000; Courtois et al., 2017) and lead to serious ecological and evolutionary consequences (Li et al., 2009; Ehrenfeld, 2010; He et al., 2020; Ren et al., 2021), including loss of biodiversity and alteration of the structure and function of the invaded ecosystem (Schirmel et al., 2016; Osborne and Gioria, 2018; Stephanie et al., 2018; Vaz et al., 2018; Giulio et al., 2022). Coastal wetland ecosystems are one of the habitats most vulnerable to alien species invasion (Zedler and Kercher, 2004; Bradley et al., 2010; Gillard et al., 2021). *Spartina alterniflora* Loisel. is a common invasive plant occurring in coastal salt marsh ecosystems (Daehler and Strong, 1996; Takahashi et al., 2019), the invasion range of which includes the coastal areas of China, France, the United Kingdom, Spain, Australia, New Zealand, and South Africa (Riddin et al., 2016; Meng et al., 2020; Yan et al., 2022). Efforts have been made worldwide to reduce the impact of *S. alterniflora* invasion and to restore local ecosystems (Diefenderfer et al., 2018; Zhao et al., 2019; Chen et al., 2020).

Wetland ecological restoration engineering is an important field that focuses on restoring wetland ecology and improving its ecological structure, processes, and functions (Daily et al., 2009; Glenn et al., 2013; Davies et al., 2014; Xie et al., 2021). Wetland ecological water replenishment has significantly enhanced the regulation and support of plant species in wetland ecosystems (Cui et al., 2009; Restrepo and Cantera, 2013; Demarco et al., 2022). In coastal wetlands, the introduction of freshwater facilitates the biodiversity restoration in addition to ecological replenishment (Proosdij et al., 2010; Cui et al., 2018; Yang et al., 2019; Huang et al., 2020). Ecological management practices have shifted the focus from removing the invasive species alone to monitoring and restoring the entire ecosystem (Zavaleta et al., 2001; Remm et al., 2019). In wetland ecosystem monitoring, plant communities are among the main indicators of invasion in comparative studies before and after restoration (Giljohann et al., 2011; Nicol et al., 2017; Arnoldi et al., 2022). Therefore, it is essential to investigate the characteristics of the coastal wetland plant communities invaded by *S. alterniflora* before and after restoration.

*S. alterniflora* has been studied extensively as an invasive species; however, most of the studies have focused on the impact of *S. alterniflora* invasion on native species in coastal wetlands without ecological restoration measures (Minchinton et al., 2006; Gerwing et al., 2021; Yue et al., 2021). Some studies have found that freshwater introduction leads to more obvious community zonality of coastal wetlands (Cui et al., 2018). After the introduction of freshwater, the diversity of plant communities first increased and then decreased, whereas the dominant salt-tolerant and halophytic plant species were gradually replaced by glycophytic plants (Cui et al., 2009; Duarte et al., 2015). Studies on the long-term invasion of *S. alterniflora* have focused on quantifying the temporal dynamics where the spread of S. alterniflora and subsequent elimination of native species was monitored over a period of time (Tang et al., 2012; Liu et al., 2017; Zhang et al., 2020; Yan et al., 2021). However, few studies have focused on the effects of freshwater introduction measures on plant diversity and interspecific associations in coastal wetlands invaded by *S. alterniflora*.

*S. alterniflora* was first artificially introduced in Yancheng, China, in 1979 (Li et al., 2005). After long-term expansion, the coastal wetlands of Yancheng currently experience a significant biological invasion by *S. alterniflora* (Gu et al., 2018; Meng et al., 2020; Zuo et al., 2021). To preserve the biodiversity and to control *S. alterniflora* invasion in the region, the Chinese government has been implementing ecological hydrological engineering, including freshwater introduction, in Dafeng Père David's Deer National Nature Reserve since 2011 (Yan et al., 2021). In this study, we documented the plant community composition in the region by *in situ* investigation in 2008 and 2018. To assess the possible consequences of freshwater introduction on the plant communities invaded by *S. alterniflora*, we analyzed species diversity and interspecific associations at the study sites. The results of this study will help to develop strategies for controlling of *S. alterniflora* invasion and improve the management of coastal wetland ecosystem biodiversity.

## Materials and methods

### Study sites

**Figure 1:**
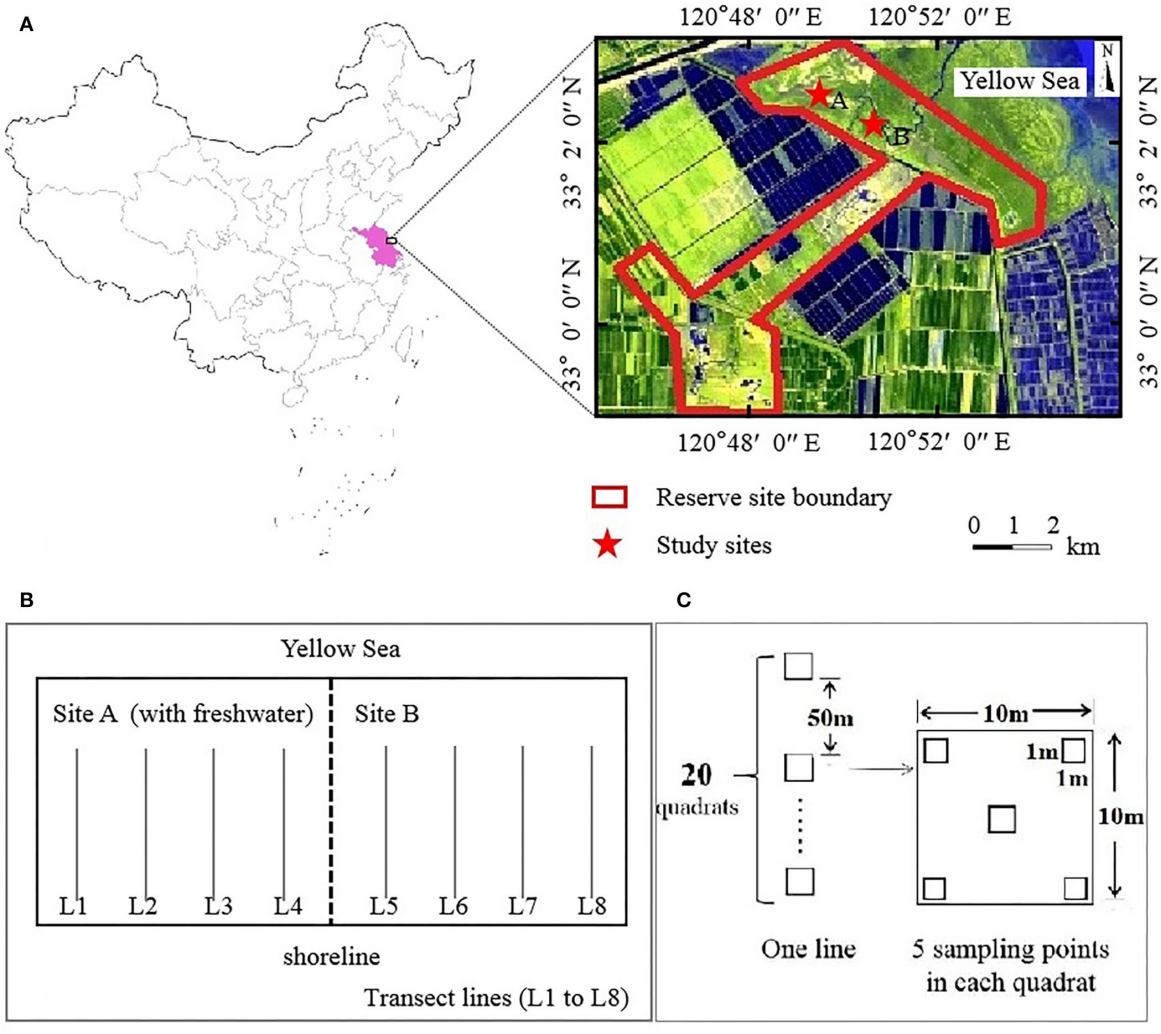
Location of the study sites. Reserve site **(A)**, transect lines design **(B)**, quadrat design **(C)**.

Dafeng Père David's Deer (*Elaphurus davidianus*) National Nature Reserve (32°58′-33°03′N, 120°47′-120°53′E) is a Ramsar site located in the Yellow Sea in the eastern part of Yancheng City, Jiangsu Province, China (Figure 1A), covering an area of 26.67 km^2^. This region experiences a marine monsoon climate and belongs to the transition zone between the north subtropical and a warm temperate zone; it is characterized by an average annual precipitation of 1,068 mm and an average annual temperature of 14.1°C (Zuo et al., 2021; Yan et al., 2022).

Through the central wetland subsidy project, ecological hydrological engineering measures were implemented by the government in the coastal wetland (third core) area of the reserve. In 2011, a dyke with a length of approximately 2.3 km was built in the middle of the third core area (Yan et al., 2021), dividing the third core area into sites A and B (Figure 1A). Freshwater was introduced into site A, whereas site B remained in its natural state. Site A was not completely enclosed and thus could still be affected by high tidal action. To enhance the restoration effect of freshwater introduction, a 10 km artificial freshwater ditch was built at site A in 2016. In summary, site A had a continuous influx of freshwater, which is occasionally affected by seawater. Site B was maintained in a tidal-action environment.

### Field investigation

Eight parallel transect lines (L1–L8) running perpendicular to the shoreline were marked for sampling at the study sites A and B; four transect lines were used per study site (Figure 1B). All parallel transect lines had 20 quadrats (Q01–Q20, with an area of 10 × 10 m) with a 50 m gap between each quadrat. Five uniformly spaced sampling points with an area of 1 m × 1 m were marked within each quadrat (Figure 1C). The quadrat was named as L×Q××. The plant species, total coverage, partial coverage, plant height, and number of plants (clumps) were recorded in each quadrat. Based on the changes in the dominant species, we estimated growth gradients among plant communities extending from the shoreline to nearshore. Two surveys were conducted in mid-August 2008 (before the introduction of freshwater) (completed by engineers working in the reserve) and mid-August 2018 (after the introduction of freshwater).

### Statistical analysis

The importance value (*IV*) was calculated to characterize the wetland plant communities. Hierarchical clustering analysis was used to study the community types of coastal wetland vegetation (Lemein et al., 2017; Gaberščik et al., 2018). The following four indices were calculated to assess the species diversity: species richness (*S*), Shannon–Wiener index (*H*), Simpson's diversity index (*D*), and Pielou evenness index (*J*). The differences in the species diversity indices between different study sites were analyzed using one-way analysis of variance with the level of significance defined at 0.05. The interspecific relationship of the dominant species was analyzed using the dominant species pair correlation test and the degree of inter-species association (Stachowicz, 2001; Turnbull et al., 2004). Between-habitat diversity (beta diversity, β) and principal coordinate analysis (PCoA) (Bray–Curtis coefficient was used as the similarity index) were used to compare the community composition of different habitats (Costa et al., 2011). The distribution and changes in the communities were analyzed by calculating the ratio of the number of each vegetation type to the total number of quadrats. All the above parameters were computed using IBM SPSS Statistics (version 22.0; SPSS Inc., Chicago, IL, USA) and R (version 3.5.2, R Foundation for Statistical Computing).


$$\begin{array}{l} IV=(\text{relative density}+\text{relative coverage}\\ \;+\text{ relative frequency})/3\\ \;H=-\sum_{i=1}^{s}P_{i}{\text{log}}_{2}P_{i}\\ \;D=1-\sum_{i=1}^{s}\frac{N_{i}\left(N_{i}-1\right)}{N\left(N-1\right)}\\ \;J=\frac{-\sum P_{i}\text{ln}P_{i}}{\text{ln}S} \end{array}$$

where *S* is the number of species (mean among quadrats), *P*_*i*_ is the ratio of individuals of species *i* to total individuals of all species, *N*_*i*_ is the number of individuals of species *i*, and *N* is the individual number of all species in the community.

The interspecific relationship of the dominant species was assessed using the dominant species pair correlation test—chi-square test (Table 1).


$$\begin{array}{l} x^{2}=\frac{N{\left(|ad-bc|-\frac{N}{2}\right)}^{2}}{\left(a+b\right)\left(c+d\right)\left(a+c\right)\left(b+d\right)} \end{array}$$

**Table 1:** Determination of inter-species association 2×2 contingency table.

		**Species B**		
**Species**		**Occurring (1)**	**Not occurring (0)**	**Statistics**
**Species A**	**Occurring (1)**	a	b	a+b
	**Not occurring (0)**	c	d	c+d
	**Statistics**	a+c	b+d	N=a+b+c+d

where *ad*- *b*c > 0, the two species are positively related; *ad*- *bc* < 0, the two species are negatively related; and *ad*- *bc* = 0, the two species are independent of each other. If *x*^2^ > 6.635, the connection between the two species is extremely significant; if 3.841 < *x*^2^ < 6.635, the connection between the two species is significant; and if *x*^2^ < 3.841, the connection between the two species is not significant.

The degree of inter-species association was calculated using the association coefficient (AC), as follows:


$$\begin{array}{l} \;ad\;\geq \;bc,then\;AC=(ad-bc)/[(a+b)(b+d)],\\ ad<bc,and\;d\;\geq \;a,then\;AC=(ad-bc)/[(a+b)(a+c)],\\ \;ad<bc,and\;d<a,then\;AC=(ad-bc)[(b+d)(d+c)], \end{array}$$

where AC represents the strength of inter-species association. An AC value closer to 1 represents a strong positive association between two species, whereas that close to −1 indicates a negative association; for AC = 0, the two species appear alone.

The between-habitat diversity index was calculated using the β_*jk*_, as follows:


$$\begin{array}{l} \beta_{jk}=\frac{e+f}{2d+e+f} \end{array}$$

where *d* is the number of species shared by the two quadrats, *e* is the number of species present in quadrat *j* but not in quadrat *k*, and *f* is the number of species present in quadrat *k* but not in quadrat *j*.

## Results

### Characteristics and changes of species composition

**Figure 2:**
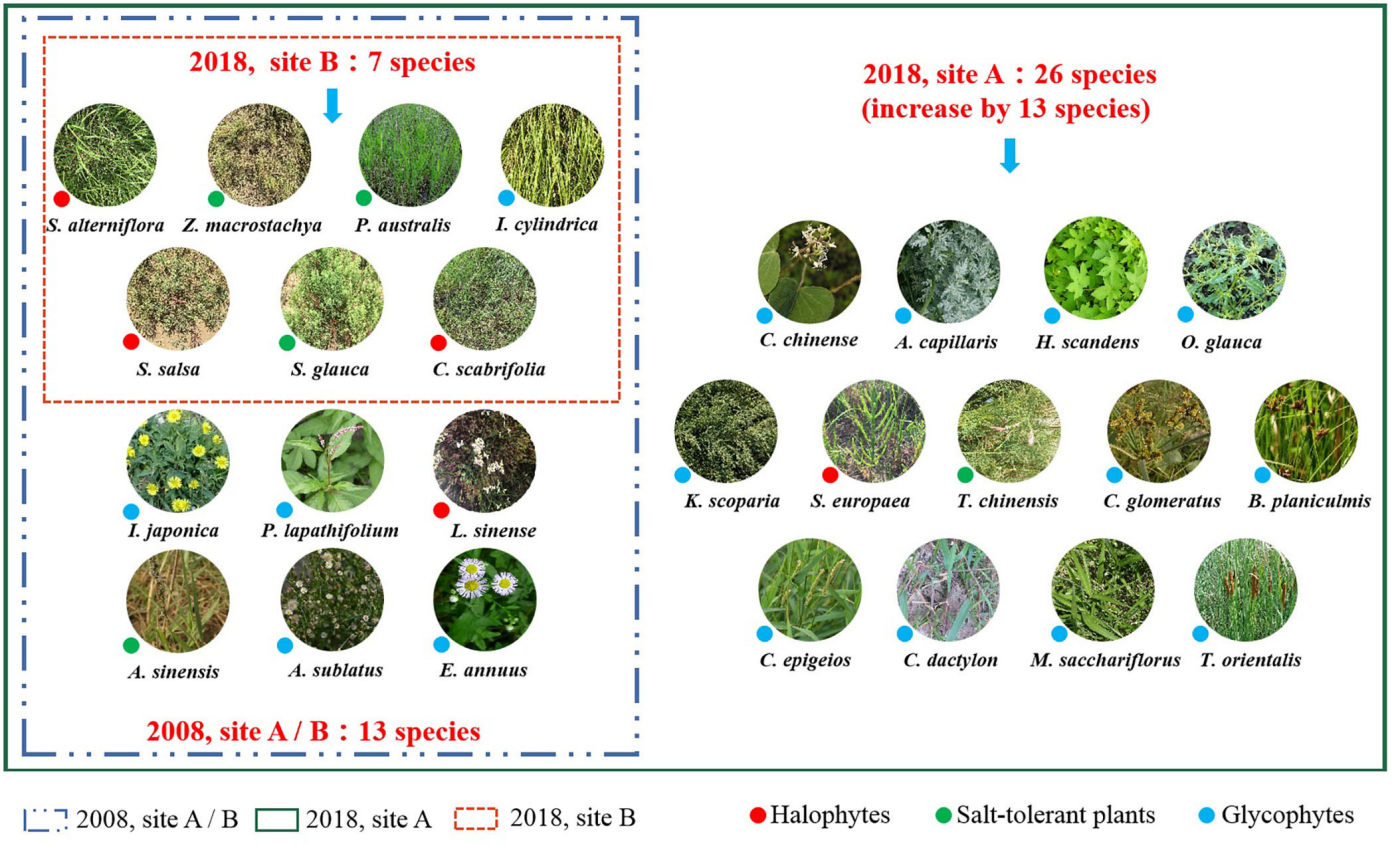
Differences in species composition between 2008 and 2018.

The field investigation in 2008 performed before the introduction of freshwater recorded 13 species, belonging to five families and 12 genera of site A; similar results were observed in site B (Figure 2). All plants were herbaceous angiosperms comprising four halophytes, four salt-tolerant species, and five glycophytes. Poaceae species were the most common, and they included *S. alterniflora, Zoysia macrostachya* Franch. et. Sav., *Phragmites australis* Trin., *Imperata cylindrica* (L.) P. Beauv., and *Aeluropus sinensis* (Debeaux) Tzvelev. Three species belonged to Asteraceae [*Aster subulatus* (Michx.) G. L. Nesom, *Erigeron annuus* (L.) Pers., and *Ixeris japonica* (Burm. f.) Nakai], two species were from Amaranthaceae [*Suaeda salsa* (L.) Pall. and *Suaeda glauca* (Bunge) Bunge], and Polygonaceae, Plumbaginaceae, and Cyperaceae were each represented by one species [*Persicaria lapathifolium* (L.) S. F. Gray, *Limonium sinense* (Girard) Kuntze, and *Carex scabrifolia* Steud., respectively].

In 2018, 26 species were identified in site A, which is a significant increase in species number from that observed in 2008. The 26 species belonged to eight families and 25 genera, including those identified in 2008. The 13 newly species identified in 2018 were included in Apocynaceae (*Cynanchum chinense* R. Br.), Asteraceae (*Artemisia capillaris* Thunb.), Cannabaceae [*Humulus scandens* (Lour.) Merr.], Amaranthaceae [*Oxybasis glauca* (L.) S. Fuentes, Uotila & Borsch, *Kochia scoparia* (L.) Schrad., and *Salicornia europaea* L.], Tamaricaceae (*Tamarix chinensis* Lour.), Cyperaceae [*Cyperus glomeratus* L. and *Bolboschoenus planiculmis* (F. Schmidt) T. V. Egorova], Poaceae [*Calamagrostis epigejos* (L.) Roth, *Cynodon dactylon* (L.) Persoon, and *Miscanthus sacchariflorus* (Maxim.) Benth. & Hook. f. ex Franch.], and Typhaceae (*Typha orientalis* C. Presl). After the introduction of freshwater, the number of halophytes and salt-tolerant plants increased from four to five, and the number of glycophytes increased from five to 16 (Figure 2).

In site B in 2018, seven species belonging to three families and six genera were identified. Four species belonged to Poaceae (*S. alterniflora, P. australis, I. cylindrica*, and *A. sinensis*), two species belonged to Amaranthaceae (*S. salsa* and *S. glauca*), and one species belonged to Cyperaceae (*C. scabrifolia*). The species composition of the wetland plant communities in site B was relatively simpler compared to that measured in 2008. After the distribution of *S. alterniflora* expanded, the number of halophytes and salt-tolerant species decreased by one species (*L. sinense* and *A. sinensis*, respectively), and the number of glycophytes decreased by four species (*I. japonica, P. lapathifolium, A. subulatus*, and *E. annuus*) (Figure 2).

### Changes in types of plant communities

**Figure 3:**
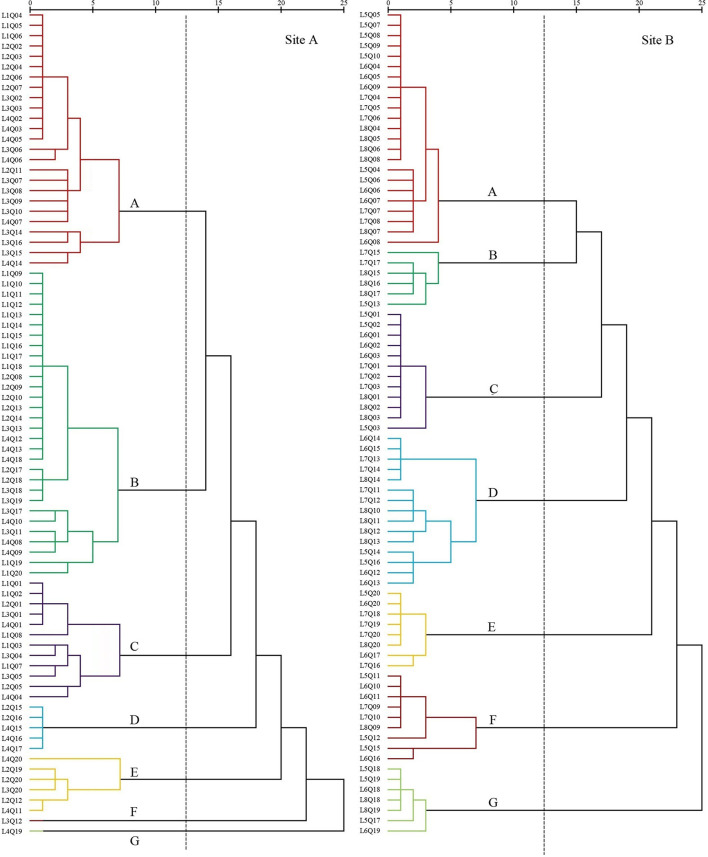
Dendrogram of coastal wetland vegetation based on cluster analysis of data collected in 2008.

The plant communities were categorized according to the importance values (*IV*) of the species within each quadrat. In 2008, seven settlement clusters (Figure 3) were identified: *S. salsa* (site A, 30%; site B, 28.75%), *P. australis* (site A, 22.5%; site B, 7.5%), *S. alterniflora* (site A, 15%; site B, 15%), *Z. macrostachya* (site A, 12.5%; site B, 18.75%), *I. cylindrica* (site A, 7.5%; site B, 10%), *C. scabrifolia* (site A, 7.5%; site B, 11.25%), and *A. sinensis* (site A, 5%; Site B, 8.75%).

The first settlement cluster (A) contained 48 quadrats (site A, 25; site B, 23). *S. salsa* was the dominant species in this community. There were 28 quadrats (site A, 13; site B, 15) in which *S. salsa* was the only species detected. There were nine quadrats (site A, 2; site B, 7) of *P. australis* associated with *S. salsa* and seven quadrats (site B) of *S. glauca* associated with *S. salsa*. The remaining five quadrats (site A, 4; site B, 1) contained the association of *P. lapathifolium, A. subulatus*, and *L. sinense*. The community represented in cluster (A) was located far from the nearshore environment at an average distance of 800 m.

The second settlement cluster (B) consisted of 36 quadrats (site A, 30; site B, 6). *P. australis* was the dominant species in the community, with 19 quadrats (site A) containing only *P. australis*. Four quadrats (site A, 3; site B, 1) contained *P. australis* associated with *S. glauca*, and nine quadrats (site A, 5; site B, 4) included *P. australis* associated with *S. salsa* or *I. cylindrica*. The remaining three quadrats (site A, 2; site B, 1) contained associations of *E. annuus, A. subulatus, I. japonica*, and *Z. macrostachya*. Settlement cluster (B) was located proximally to the nearshore.

The third settlement cluster (C) consisted of 24 quadrats (site A, 12; site B, 12). *S. alterniflora* was the dominant species in the community, and 16 quadrats (site A, 5; site B, 11) contained only *S. alterniflora*. The other eight quadrats (site A, 7; site B, 1) included *S. salsa, S. glauca, C. scabrifolia*, and *P. australis*. Settlement cluster (C) was located farthest from the nearshore of all the defined groups.

The fourth settlement cluster (D) included 20 quadrats (site A, 5; site B, 15). *Z. macrostachya* was the dominant species in the community, with 10 quadrats (site A, 5; site B, 5) of *Z. macrostachya* as a single species. The other 10 quadrats (site B) contained the association of *S. salsa, S. glauca*, and *C. scabrifolia*. This community was centrally located in the sample line.

The fifth settlement cluster (E) contained 14 quadrats (site A, 6; site B, 8). *I. cylindrica* was the dominant species in the community, and eight quadrats (site A, 2; site B, 6) contained only *I. cylindrica*. The remaining six quadrats (site A, 4; site B, 2) contained an association of *P. australis, C. scabrifolia*, and *L. sinense*. This community was located closest to the nearshore.

The sixth settlement cluster (F) included 10 quadrats (site A, 1; site B, 9). *C. scabrifolia* was the dominant species in the community, accounting for seven single-species quadrats (site A, 1; site B, 6). The remaining three quadrats (site B) were associated with *I. cylindrica* and *S. salsa*. This community was centrally located in the sample line.

The seventh settlement cluster (G) contained eight quadrats (site A, 1; site B, 7). *A. sinensis* was the dominant species in the community, and six quadrats (site A, 1; site B, 5) contained only *A. sinensis*. The other two quadrats (site B) were *A. sinensis* associated with *I. cylindrica* and *S. salsa*. This community was located proximally to the nearshore. The average distance is less than 100 m.

**Figure 4:**
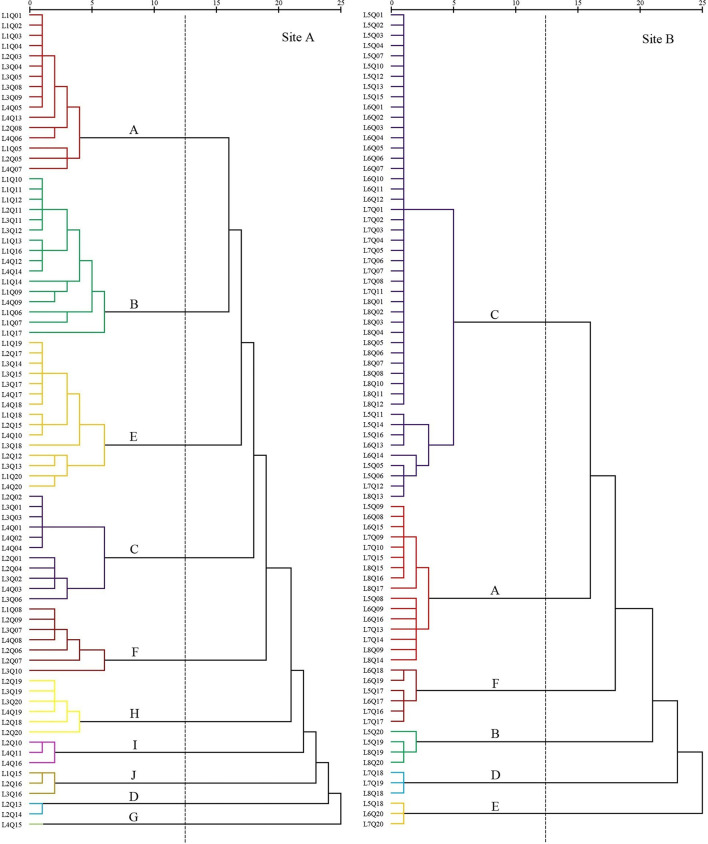
Dendrogram of coastal wetland vegetation based on cluster analysis of data collected in 2018.

In site A in 2018, freshwater introduction resulted in 10 settlement cluster (Figure 4) with dominant species including *S. salsa* (20%), *P. australis* (20%), *S. alterniflora* (13.75%), *Z. macrostachya* (2.5%), *I. cylindrica* (18.75%), *C. scabrifolia* (8.75%), *A. sinensis* (1.25%), *M. sacchariflorus* (7.5%), *T. chinensis* (3.75%), and *S. europaea* (3.75%).

The first settlement cluster (A) included 16 quadrats. *S. salsa* was the dominant species in the community. There were 10 quadrats containing only *S. salsa*, accounting for 63%. The remaining six quadrats were *S. salsa* associated with *S. europaea, P. lapathifolium, P. australis*, and *L. sinense*. This community was located far from the nearshore.

The second settlement cluster (B) contained 16 quadrats. *P. australis* was the dominant species in the community. There were six quadrats containing only *P. australis*. There were four quadrats of *P. australis* associated with *I. cylindrica* and two quadrats of *P. australis* associated with *S. salsa*. The remaining four quadrats were associated with *E. annuus, A. subulatus, I. japonica, O. glauca, K. scoparia, T. orientalis*, and *Z. macrostachya*. This community was located proximally to the nearshore.

The third settlement cluster (E) included 15 quadrats. *I. cylindrica* was the dominant species in the community. Seven quadrats contained only *I. cylindrica*. The remaining eight quadrats were associated with *P. australis, C. scabrifolia, M. sacchariflorus, C. epigejos, A. capillaris, G. aparine, E. annuus, H. scandens*, and *C. chinense*. This community was closest to the nearshore.

The fourth settlement cluster (C) contained 11 quadrats. *S. alterniflora* was the dominant species in the community. Six quadrats contained only *S. alterniflora*, accounting for 55%. The remaining five quadrats were associated with *S. salsa, S. glauca*, and *C. scabrifolia*. This community was located the farthest from the nearshore.

The fifth settlement cluster (F) contained seven quadrats. *C. scabrifolia* was the dominant species in the community. The remaining four quadrats were associated with *S. europaea*, accounting for 57%. The remaining three quadrats were associated with *I. cylindrica, S. europaea, B. planiculmis, C. glomeratus*, and *S. salsa*. This community was centrally located in the sample line.

The sixth settlement cluster (H) contained six quadrats. *M. sacchariflorus* was the dominant species in the community, and four quadrats contained only *M. sacchariflorus*, accounting for 67%. The remaining two quadrats were associated with *I. cylindrica* and *P. australis*. This community was close to the nearshore.

The seventh settlement cluster (I) included three quadrats. *S. europaea* was the dominant species in the community, and all quadrats included *S. salsa*. This community was close to the nearshore.

The eighth settlement cluster (J) included three quadrats. *T. chinensis* was the dominant species in the community, and all quadrats included *I. cylindrica* and *S. salsa*. This community was close to the nearshore.

The ninth settlement cluster (D) consisted of two quadrats. *Z. macrostachya* was the dominant species in the community, and all quadrats also contained *C. dactylon*. This community was located in the middle of the sample line.

The tenth settlement cluster (G) consisted of one quadrat. *A. sinensis* was the dominant species in the community, and the quadrat contained only *A. sinensis*. This community was close to the nearshore.

In site B in 2018, without freshwater introduction contained six settlement cluster (Figure 4), with dominant species including *S. salsa* (20%), *P. australis* (5%), *S. alterniflora* (60%), *C. scabrifolia* (7.5%), *I. cylindrica* (3.75%), and *Z. macrostachya* (3.75%).

The first settlement cluster (C) contained 48 quadrats. *S. alterniflora* was the dominant species in the community, and 39 quadrats contained only *S. alterniflora*, accounting for 81% of the quadrats. The other nine quadrats were associated with *S. salsa, S. glauca*, and *C. scabrifolia*. This community was farthest from the nearshore.

The second settlement cluster (A) contained 16 quadrats. *S. salsa* was the dominant species, and eight quadrats contained only *S. salsa*, accounting for 50%. There were seven quadrats of *S. alterniflora* associated with *S. salsa* and one quadrat containing *Z. macrostachya* associated with *S. salsa*. This community was far from the nearshore. This community was located in the middle of the sample line.

The third settlement cluster (F) contained six quadrats. *C. scabrifolia* was the dominant species, and two quadrats contained only *C. scabrifolia*. The other four quadrats contained *C. scabrifolia* associated with *S. salsa*. This community was close to the nearshore. The average distance is less than 150 m.

The fourth settlement cluster (B) contained four quadrats. *P. australis* was the dominant species in the community, and one quadrat contained only *P. australis*. There are three quadrats of *P. australis* associated with *I. cylindrica*. This community was very close to the nearshore.

The fifth settlement cluster (D) consisted of three quadrats. *Z. macrostachya* was the dominant species in the community, and one quadrat contained *A. sinensis* as a single species. The other two quadrats were *Z. macrostachya* associated with *C. scabrifolia* and *S. salsa*. This community was close to the nearshore. The average distance is less than 150 m.

The sixth settlement cluster (E) consisted of three quadrats. *I. cylindrica* was the dominant species in the community. There were three quadrats of *I. cylindrica* as a single species. This community was closest to the nearshore.

### Changes in plant species diversity

As shown in Table 2, species diversity in the coastal wetland plant communities was generally low. In 2018, although the plant species diversity at site A increased (based on the *S* index for settlement clusters B, C, D, E, and F; *H* index for settlement clusters B, C, E, and F; and *D* index for settlement clusters B, C, and E) following the introduction of freshwater, the overall species diversity of the coastal wetland vegetation remained low. In addition, in site B, we observed a reduction in species diversity (*S* index for settlement clusters A, B, C, and E and *H* index and *D* index for settlement clusters C and E) without freshwater introduction compared with that in 2008.

**Table 2:** Species diversity of plant communities in coastal wetlands invaded by *S. alterniflora*.

	***S***				***H***				***D***				***J***			
**Settlement cluster**	**2008**		**2018**		**2008**		**2018**		**2008**		**2018**		**2008**		**2018**	
	**Site A**	**Site B**	**Site A**	**Site B**	**Site A**	**Site B**	**Site A**	**Site B**	**Site A**	**Site B**	**Site A**	**Site B**	**Site A**	**Site B**	**Site A**	**Site B**
A (*S. salsa*)	1.52 ± 0.54 ns	1.52 ± 0.48 ns	1.41 ± 0.49 *	1.50 ± 0.50 ns	0.20 ± 0.24 ns	0.21 ± 0.22 ns	0.15 ± 0.18 *	0.45 ± 0.46 *	0.12 ± 0.15 ns	0.12 ± 0.12 ns	0.08 ± 0.11 *	0.28 ± 0.29 *	0.84 ± 0.19 ns	0.86 ± 0.17 ns	0.80 ± 0.25 *	0.95 ± 0.07 *
B (*P. australis*)	1.70 ± 0.82 ns	1.65 ± 0.82 ns	1.81 ± 0.75 *	1.63 ± 0.43 ns	0.29 ± 0.32 ns	0.27 ± 0.35 ns	0.33 ± 0.28 *	0.67 ± 0.39 *	0.19 ± 0.21 ns	0.18 ± 0.18 ns	0.21 ± 0.18 ns	0.43 ± 0.25 *	0.92 ± 0.11 ns	0.89 ± 0.14 ns	0.84 ± 0.17 *	0.95 ± 0.04 ns
C (*S. alterniflora*)	1.33 ± 0.47 ns	1.33 ± 0.47 ns	1.45 ± 0.50 *	1.17 ± 0.37 *	0.19 ± 0.27 ns	0.18 ± 0.32 ns	0.23 ± 0.25 *	0.12 ± 0.27 *	0.13 ± 0.18 ns	0.16 ± 0.17 ns	0.15 ± 0.16 ns	0.11 ± 0.26 ns	0.94 ± 0.09 ns	0.97 ± 0.10 ns	0.88 ± 0.13 *	0.95 ± 0.11 ns
D (*Z. macrostachya*)	1.28 ± 0.45 ns	1.50 ± 0.50 ns	1.50 ± 0.50 ns	2.00 ± 0.82 *	0.30 ± 0.30 ns	0.32 ± 0.20 ns	0.14 ± 0.14 *	0.49 ± 0.40 *	0.21 ± 0.21 ns	0.23 ± 0.25 ns	0.08 ± 0.08 *	0.31 ± 0.22 *	0.94 ± 0.07 ns	0.95 ± 0.06 ns	0.71 ± 0.29 *	0.97 ± 0.03 ns
E (*I. cylindrica*)	1.41 ± 0.41 ns	1.43 ± 0.48 ns	1.80 ± 0.98 *	1.00 ± 0.00 *	0.21 ± 0.25 ns	0.19 ± 0.18 ns	0.25 ± 0.25 ns	0.00 ± 0.00 *	0.13 ± 0.16 ns	0.12 ± 0.14 ns	0.14 ± 0.13 ns	0.00 ± 0.00 *	0.88 ± 0.15 ns	0.90 ± 0.15 ns	0.76 ± 0.23 *	1.00 ± 0.00 *
F (*C. scabrifolia*)	1.00 ± 0.00 ns	1.34 ± 0.46 ns	1.43 ± 0.49 ns	1.67 ± 0.47 *	0.17 ± 0.24 ns	0.22 ± 0.25 ns	0.18 ± 0.21 ns	0.56 ± 0.39 *	0.11 ± 0.16 ns	0.11 ± 0.10 ns	0.11 ± 0.13 ns	0.41 ± 0.29 *	0.91 ± 0.12 ns	0.90 ± 0.11 ns	0.83 ± 0.19 *	0.89 ± 0.08 ns
G (*A. sinensis*)	1.00 ± 0.00 ns	1.29 ± 0.43 ns	1.00 ± 0.00 ns	—	0.14 ± 0.24 ns	0.15 ± 0.16 ns	0.00 ± 0.00 *	—	0.09 ± 0.16 ns	0.09 ± 0.15 ns	0.00 ± 0.00 *	—	0.95 ± 0.08 ns	0.98 ± 0.07 ns	1.00 ± 0.00 ns	—
H (*M. sacchariflorus*)	—	—	1.33 ± 0.47	—	—	—	0.17 ± 0.24	—	—	—	0.11 ± 0.16	—	—	—	0.91 ± 0.13	—
I (*S. europaea*)	—	—	1.33 ± 0.47	—	—	—	0.14 ± 0.20	—	—	—	0.09 ± 0.12	—	—	—	0.87 ± 0.18	—
J (*T. chinensis*)	—	—	1.33 ± 0.50	—	—	—	0.18 ± 0.26	—	—	—	0.06 ± 0.08	—	—	—	0.93 ± 0.10	—

“ns,” not significant; asterisk indicates significant differences at P < 0.05.

In 2008, there was no significant difference in the species diversity of plant communities at sites A and B. We observed differences in diversity indices of different communities (Table 2). There were no significant differences in species richness (*S*) among the communities. According to the Shannon–Wiener index (*H*) and Simpson's diversity index (*D*), the species diversity of the *S. alterniflora* and *Z. macrostachya* communities was higher than that of other communities. Each community had a higher Pielou's evenness index (*J*).

In 2018, among the 80 quadrats at site A, 43 quadrats (accounting for 53.75%) contained a single species. The *S* index of the *S. salsa* communities decreased slightly; no change was detected in the case of *Z. macrocephala* and *A. sinensis* communities, whereas *S* of the other communities increased significantly. According to the diversity indices *H* and *D*, the diversity of the *P. australis* community was higher than that of other communities. Compared with the values in 2008, the species diversity of the other three vegetation communities was not high. *J* index was high, but compared to 2008, it was reduced in all communities except that of *A. sinensis*.

In 2018, among the 80 quadrats in site B, 55 quadrats (accounting for 68.75%) comprised a single species. The *S* index of the *P. australis, Z. macrostachya*, and *C. scabrifolia* communities increased, while that of *S. salsa, S. alterniflora*, and *I. cylindrica* communities decreased. According to *H* and *D* indices, the diversity of the *S. alterniflora* and *I. cylindrica* communities decreased significantly, whereas other communities showed a significant increase in diversity. Only the *J* of *C. scabrifolia* decreased slightly and that of the other communities was >0.95, indicating a significant increase.

**Figure 5:**
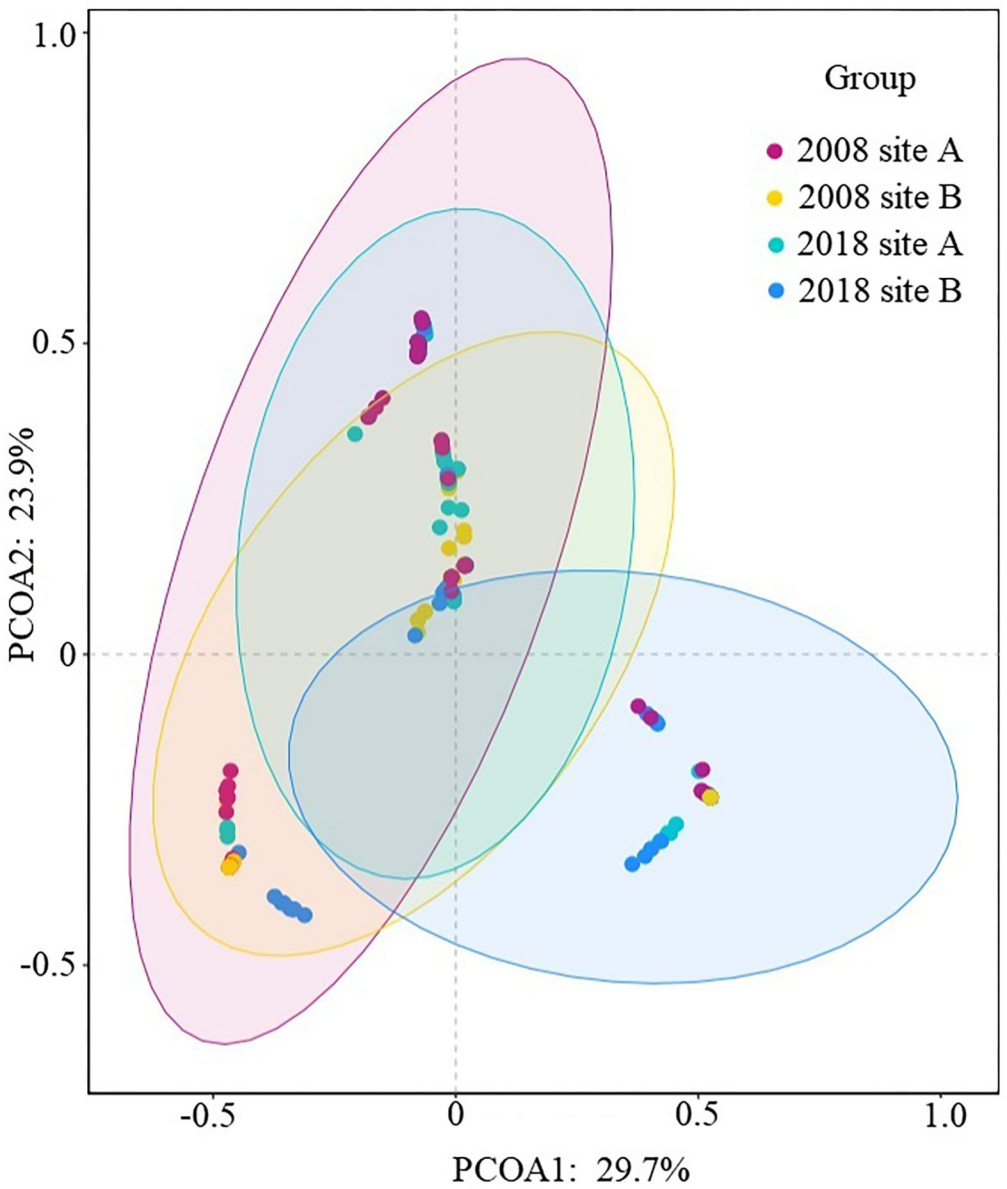
Similarity in species composition in coastal wetlands.

Analysis of between-habitat diversity values showed a value of zero between site A and site B in 2008; the value of site A between 2008 and 2018 was 0.33, the value of site B between 2008 and 2018 was 0.30, and the value between site A and site B in 2018 was 0.40. Both the introduction of freshwater and further invasion by *S. alterniflora* resulted in the replacement of native community, with the introduction of the freshwater showing a faster replacement rate. Analysis of similarity in species composition in the coastal wetlands (Figure 5) showed that the plant species composition in site B in 2018 was markedly separated from that of the other three groups, thus indicating a different species composition.

### Growth gradient among plant communities

The dominant species in the coastal wetland vegetation can be divided into three types, namely halophytes, salt-tolerant plants, and glycophytes, as it extends from the shoreline to the nearshore. *S. salsa, S. alterniflora, C. scabrifolia, S. europaea*, and *L. sinense* are halophytes. *P. australis, Z. macrostachya, T. chinensis, S. glauca*, and *A. sinensis* are salt-tolerant plants, and the other species such as *I. cylindrica, A. sublatus, E. annuus, I. japonica, P. lapathifolium*, and *M. sacchariflorus* are glycophytes.

**Figure 6:**
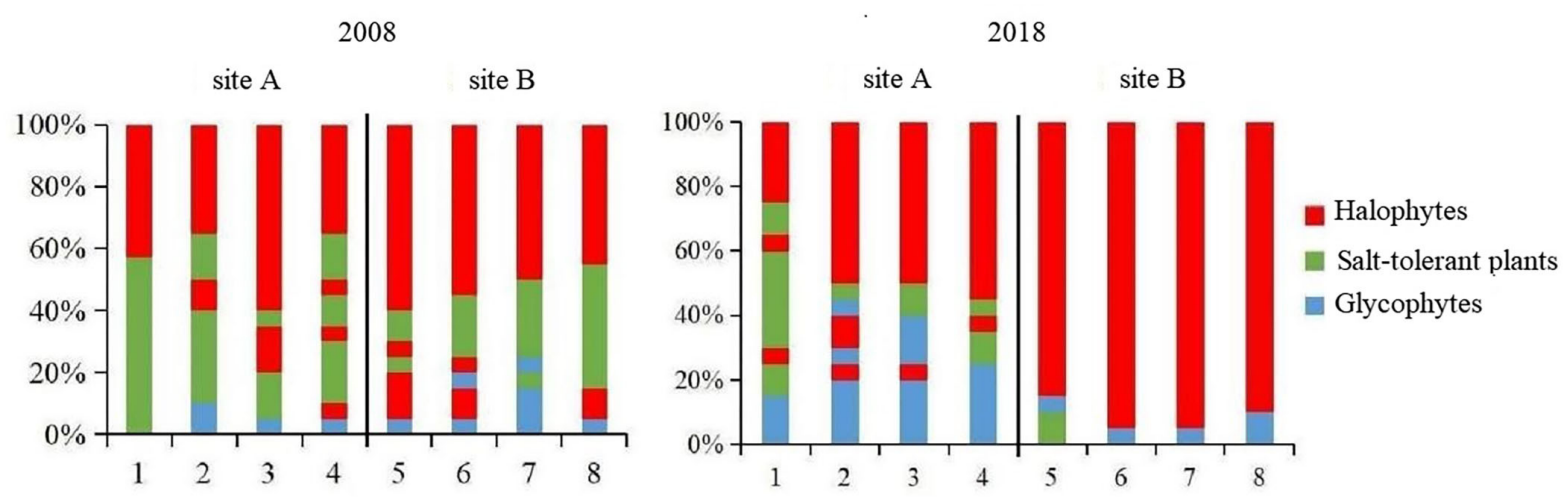
Distribution of vegetation types in coastal wetlands.

The plant communities between 2008 and 2018 in the region with freshwater introduction (site A) were compared (Figure 6). The glycophytic plant communities were distinctly enlarged. There was no evident change in the halophyte community; the habitat of salt-tolerant plants appeared compressed. In a case of the vegetation in the area without freshwater introduction (site B), because of the further expansion of *S. alterniflora* (Figure 6), the halophyte community was significantly expanded, whereas the habitats of the glycophytic and salt-tolerant plant communities were compressed.

**Figure 7:**
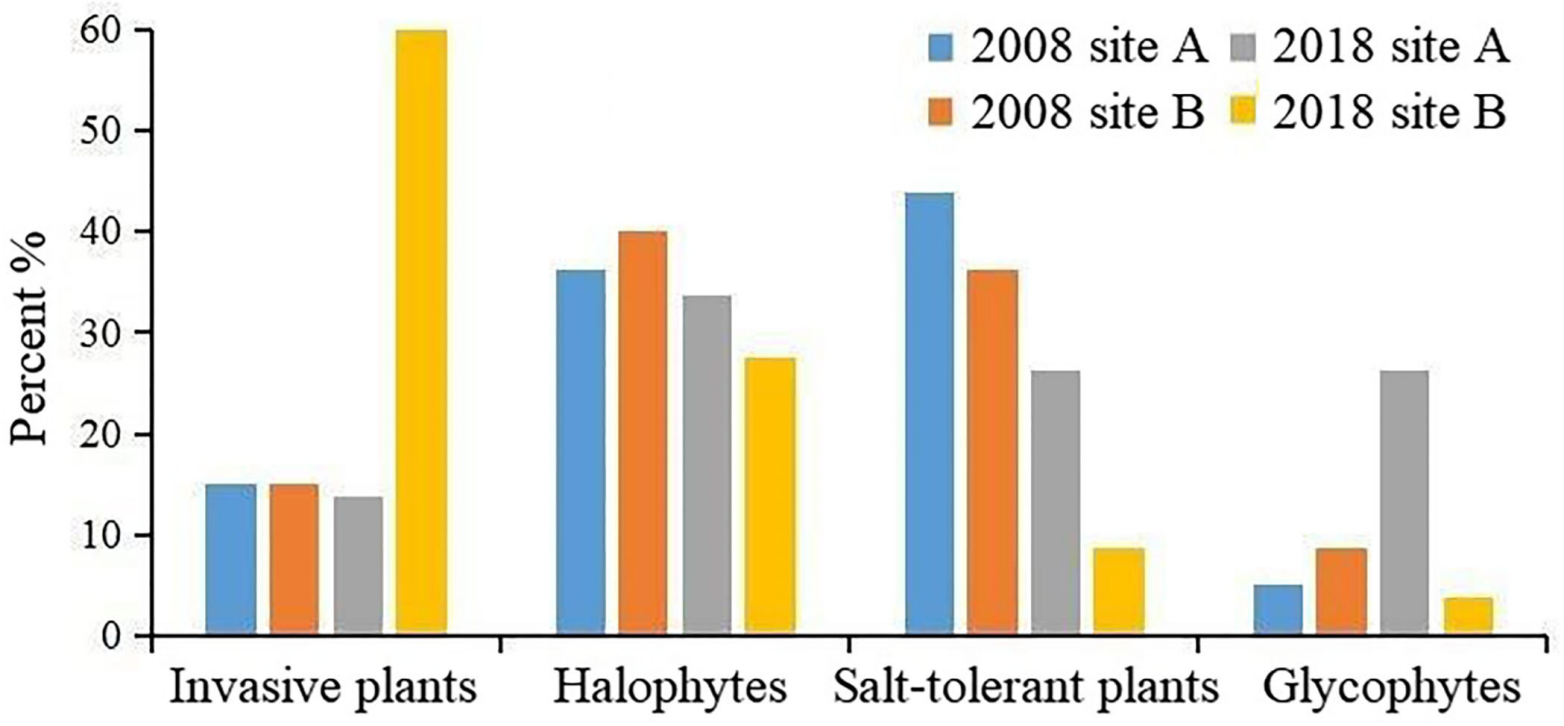
Changes in the proportion of coastal wetland vegetation types.

The proportion of samples with different vegetation types between 2018 and 2008 were analyzed (Figure 7). The proportion of invasive plants increased in areas without freshwater introduction (site B), whereas the proportion of glycophytes increased in areas with freshwater introduction (site A).

### Changes in interspecific relationships among species

**Figure 8:**
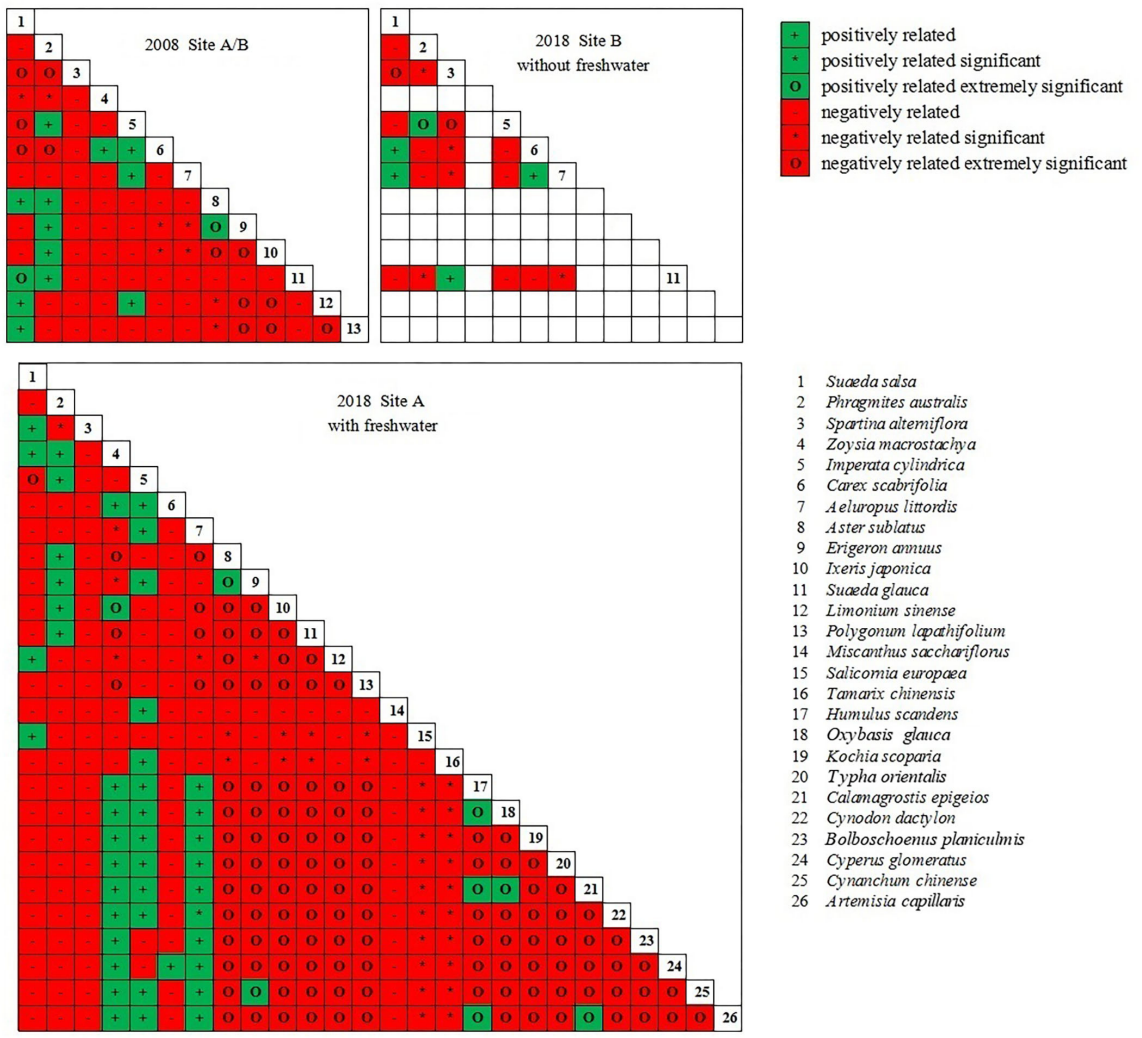
Semi-matrix for chi-square test of dominant species.

The dominant species pair correlation test showed a significant negative correlation among the plant communities in the coastal wetland invaded by *S. alterniflora* (Figure 8); the positive–negative association ratio was less than 1. In site A, the ratio increased in 2018 (0.31) compared to that in 2008 (0.22) after the introduction of freshwater. In site B, the ratio decreased in 2018 (0.19) compared to that in 2008 (0.22) following the continued invasion of *S. alterniflora*.

In site A, the introduction of freshwater significantly altered the interspecific relationships of *S. salsa* with other species. For example, we observed a shift in the interspecific relationships among *S. salsa, S. alterniflora*, and *Z. macrostachya* from negative to positive. The interspecific relationships among *S. salsa, S. glauca, A. subulatus*, and *P. lapathifolium* shifted from positive to negative. In site B (without freshwater introduction), the interspecific relationships of native halophytes (*S. salsa, C. scabrifolia*, and *Z. macrostachya*) changed from negative to positive or positive to negative. The interspecific relationships between the glycophytes *A. sublatus* and *S. salsa, P. australis*, and *S. alterniflora* shifted from negative to positive.

**Figure 9:**
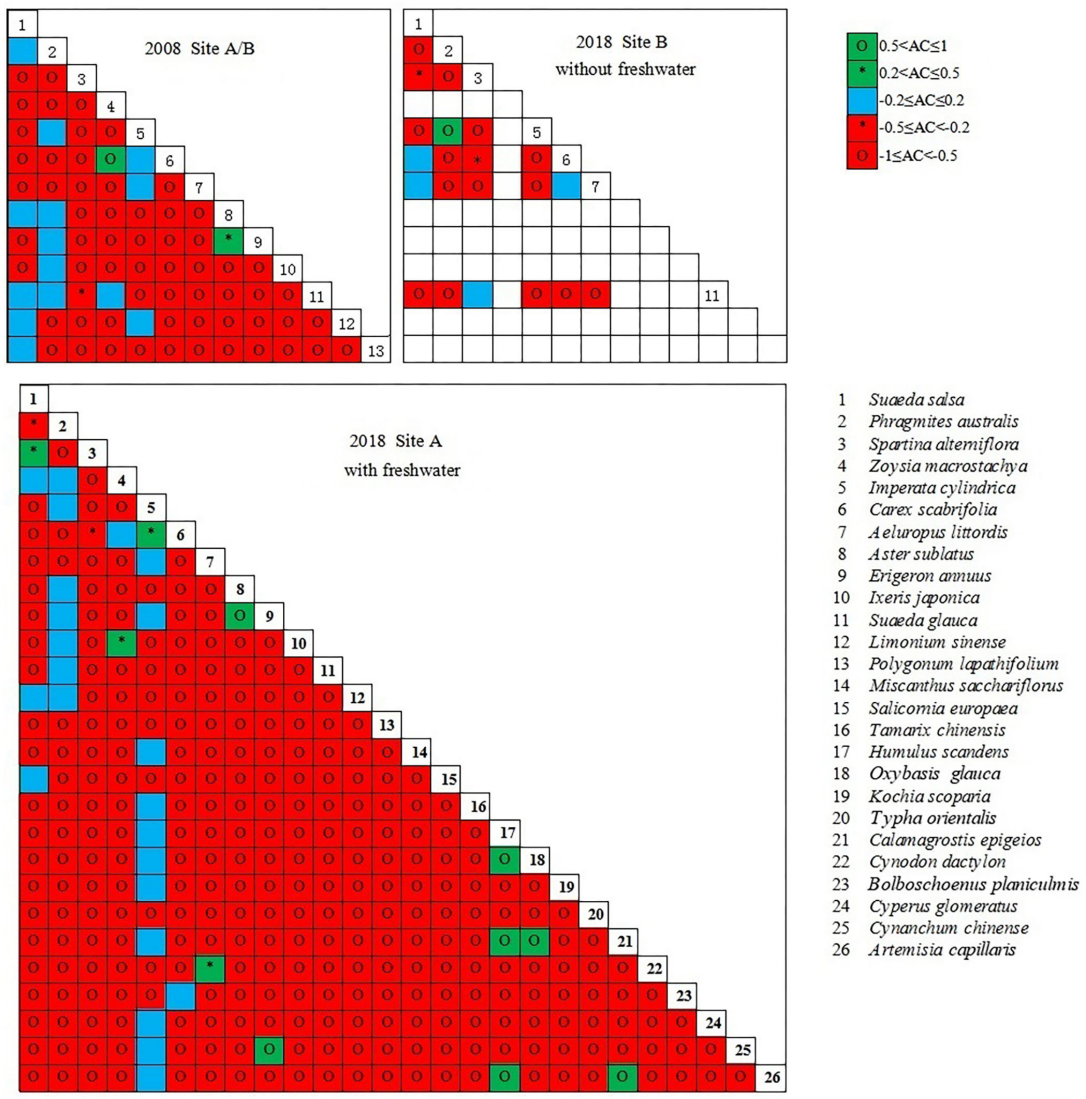
Dominant species AC value semi-matrix.

Determination of the degree of inter-species association revealed a stronger negative association among plant communities in the coastal wetlands invaded by *S. alterniflora* (Figure 9). The results indicate that that the probability of two species appearing in the same habitat is low and that there is usually a negative correlation or no obvious interspecific relationship between two species. In site A, freshwater introduction reduced the strength of the negative associations between species. In site B, further expansion of *S. alterniflora* increased the strength of the negative associations between species; most species in the communities were relatively independent.

## Discussion

### Effects of *S. alterniflora* invasion on coastal wetland vegetation

*S. alterniflora* is one of the most dominant invasive species in coastal wetlands of China (Liu et al., 2020; Meng et al., 2020). Unless control measures are implemented, *S. alterniflora* would cause damage to coastal wetland ecosystems and negatively affect their biodiversity. Following the invasion of *S. alterniflora*, the coastal wetland plant communities in the 2008 investigation comprised dominant species and a few associated species with low species diversity, ecological dominance, and community evenness. In site B, where *S. alterniflora* further expanded in 2018, the species composition and types of plant communities appeared simpler than those in 2008. In addition to the relatively simple habitat conditions of the coastal wetland, the species composition of the quadrats was modest, and the associated species were limited owing to the influence of the invasive plant (Lemein et al., 2017; Li et al., 2020).

The niche theory states that the construction of a community is the result of species interactions under the pressure of a specific environment. Interspecific relationships are closely related to the degree of niche overlap during the process of colonization and coexistence in local habitats. Native plants in the invasive coastal wetland community of *S. alterniflora* were negatively correlated; this is an indication that the community in this area is unstable, immature, and prone to fluctuations.

The distribution of coastal wetland vegetation from the shorelines to nearshore showed evident changes in the species composition (Lemein et al., 2017), including *S. alterniflora, S. salsa, C. scabrifolia, S. salina, Z. macrostachya, T. chinensis, P. australis, A. sinensis, I. cylindrica*, and *M. sacchariflorus* communities. In 2018, in site B, where there was no influx of freshwater, we observed an increase in the growth expansion of *S. alterniflora*. According to the proportion of samples with different vegetation community types between 2018 and 2008, the survival pressure of native salt-tolerance plants was the greatest in coastal wetlands invaded by *S. alterniflora*, which significantly encroached the growth space of the native vegetation. This resulted in a sharp decrease or even the disappearance of certain native species. The biogeomorphic feedback of invasive species can alter the selection pressure of local species at the population and community levels, leading to changes in the evolutionary process (Wang et al., 2019; Gerwing et al., 2021).

### Effect of freshwater on plant species diversity in coastal wetlands invaded by *S. alterniflora*

Studies have shown that hydrology is a key factor for the restoration of coastal wetlands (Zhao et al., 2016; Yang et al., 2017; Gaberščik et al., 2018; Liu et al., 2021). Freshwater is an effective measure for controlling the growth of *S. alterniflora*. Similarly, our findings indicated that freshwater introduction exhibited positive effects on the diversity of plant communities in the coastal wetlands invaded by *S. alterniflora*. In 2018, the species diversity increased at the study site with the introduction of freshwater. The number of species in the coastal wetland vegetation increased from 13 to 26 species, and there was a significant reduction in the area occupied by the invasive species. After freshwater introduction, three vegetation community types were added in 2018, leading to an increase in plant species diversity. These results indicated that freshwater introduction positively influenced the native plant communities of the coastal wetlands invaded by *S. alterniflora*. Although the similarity of species composition after freshwater introduction in site A was not markedly separated from that in 2008, it was markedly separated from that in site B (without freshwater introduction) in 2018. From the perspective of species composition similarity, freshwater introduction significantly contributed to the biodiversity maintenance of plant communities in coastal wetlands invaded by *S. alterniflora*. These results highlight the importance of early intervention in controlling the spread of invasive species, such as *S. alterniflora*, in coastal wetlands.

Moreover, in 2018, we identified certain wetland plants that prefer freshwater environments (such as *T. orientalis* and *M. sacchariflora*). Future vegetation monitoring should thus follow these patterns. Although the freshwater introduction restoration project had clear positive effects on the biodiversity of the invaded coastal wetland, it had potential shortcomings that require control measures. At the freshwater introduction site, we observed an increase in species diversity in terms of glycophyte expansion. Future studies should monitor glycophytes as indicator species to determine their impact on native halophyte communities.

### Effect of freshwater on plant interspecific associations in coastal wetlands invaded by *S. alterniflora*

The results of the dominant species pair correlation test suggested that freshwater introduction did not reverse the positive–negative associations among coastal wetland plant communities. The value of the positive–negative association ratio was increased compared to that in 2008; however, it remained < 1, indicating that the vegetation in the Yancheng coastal wetlands invaded by *S. alterniflora* was unstable. Furthermore, during the same period, the positive–negative association ratio of the site where freshwater was introduced (0.31) was significantly higher than that of the site where freshwater was not introduced (0.19). Moreover, the development of the existing plant communities was in the initial stages and thus showed strong instability (Callaway, 1995; Wang et al., 2012; García-Cervigón et al., 2013). The interspecific relationships between *S. salsa* and other species were significantly altered after the introduction of freshwater. Therefore, *S. salsa* should be used as an indicator species to monitor the restoration of coastal wetlands invaded by *S. alterniflora* using freshwater introduction. Furthermore, the measurements of the degree of inter-species association suggested that freshwater introduction reduced the strength of negative associations between species. Without the introduction of freshwater, an increase in the strength of negative associations between species was observed. The introduction of freshwater thus promoted the stability of the plant communities in the coastal wetlands invaded by *S. alterniflora*.

Environmental factors, particularly soil salinity, significantly influence the distribution of vegetation (Amores et al., 2013; Telesh et al., 2013; Archer et al., 2021; Danihelka et al., 2022). Because our investigations did not account for environmental indicators such as soil, this study did not analyze the environmental impact on different vegetation communities. This study offers a preliminary judgment based on the type of vegetation, and the introduction of freshwater to the changes in soil salinity requires further analysis. Future research should include sampling and analysis of various environmental factors to explain the effects of plant invasions and coastal wetland restoration on native plant communities.

## Conclusion

This study comprehensively investigated the influence of freshwater introduction on vegetation in coastal wetlands invaded by *S. alterniflora*. There was an overall reduction in plant species diversity in the coastal wetlands invaded by *S. alterniflora*. Without freshwater introduction, *S. alterniflora* would further occupy the space of native vegetation communities, resulting in a sharp reduction or even disappearance of native species. Although freshwater introduction increased the number and diversity of plants and effectively inhibited further invasion of *S. alterniflora*, it also increased the risk of expansion of the glycophyte community. Both freshwater introduction and intensified invasion of *S. alterniflora* introduced a shift in the interspecific relationship between *S. salsa* and other species. Thus, *S. salsa* is the most important native species in Yancheng coastal wetlands. Future studies should focus on observing community changes in *S. salsa* and glycophytes by setting up fixed monitoring points to regulate the influx of freshwater in the wetlands. This study emphasizes the need for early intervention by implementing restoration measures in coastal wetlands invaded by *S. alterniflora* and calls for a follow-up monitoring and meticulous management.

## Data availability statement

The original contributions presented in the study are included in the article/supplementary material, further inquiries can be directed to the corresponding author.

## Author contributions

LC, WL, and ZD conceived the ideas and designed methodology. ZD, XZ, YC, and RY collected the data. ZD analyzed the data. ZD and YL wrote the manuscript. All authors contributed critically to the drafts and gave final approval for publication.

## Funding

This work was supported by the Project Fund Research Grant of Yellow Sea Academy of Wetland Research (20210109) and the National Key R&D Program of China (2017YFC0506200).

## Conflict of interest

The authors declare that the research was conducted in the absence of any commercial or financial relationships that could be construed as a potential conflict of interest.

## Publisher's note

All claims expressed in this article are solely those of the authors and do not necessarily represent those of their affiliated organizations, or those of the publisher, the editors and the reviewers. Any product that may be evaluated in this article, or claim that may be made by its manufacturer, is not guaranteed or endorsed by the publisher.
